# Supplementary material for: Immunopharmacological Activities of Luteolin in Chronic Diseases
Source: Int J Mol Sci. 2023 Jan 21;24(3):2136. doi: 10.3390/ijms24032136 (PMC9917216; doi:10.3390/ijms24032136)
Supplement: Supplementary file 1 [file ijms-24-02136-s001.zip › ijms-2144615-supplementary.pdf]

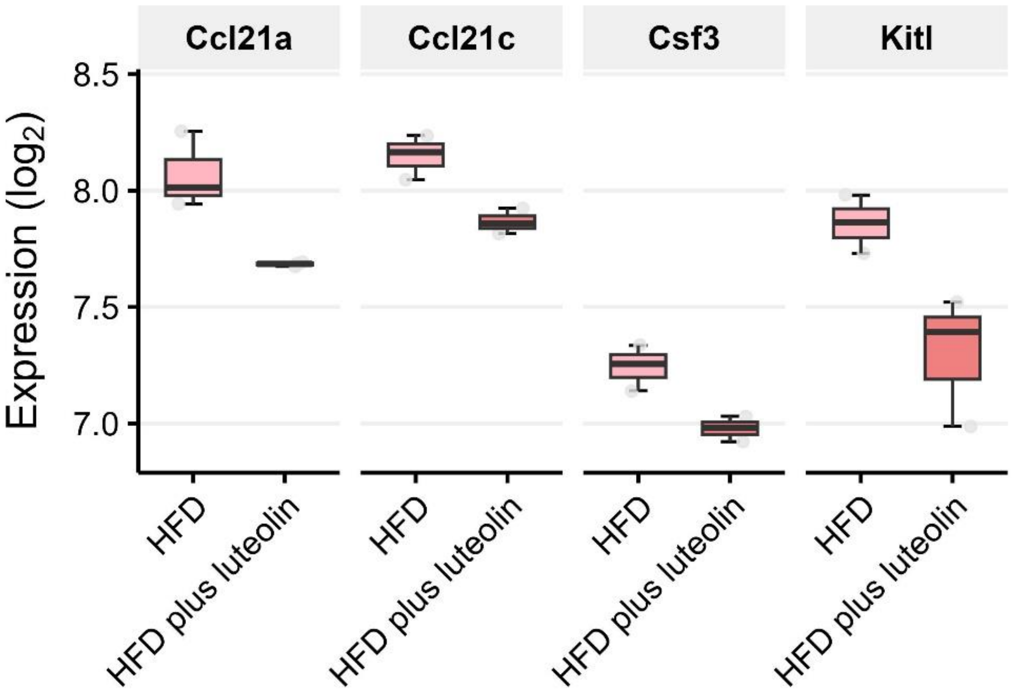

Supplementary Figure S1. GSE209778

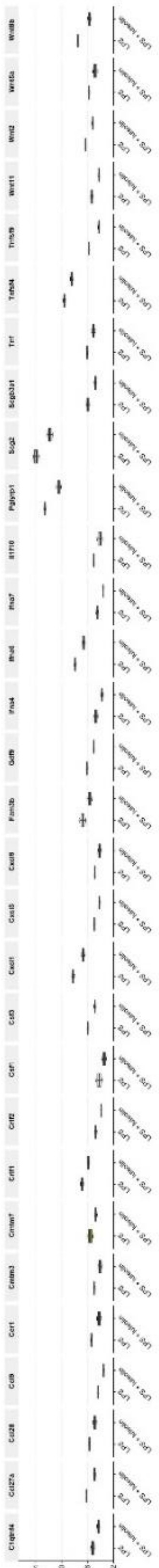

Supplementary Figure S2. GSE181522

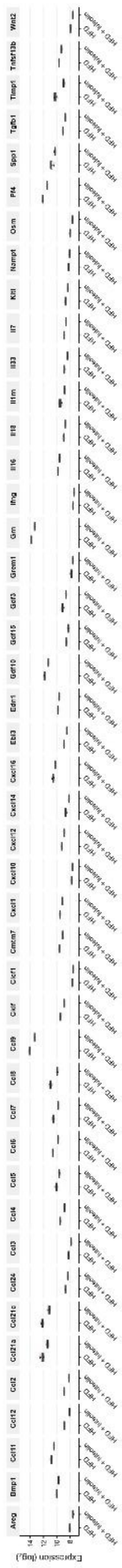

Supplementary Figure S3. GSE111412

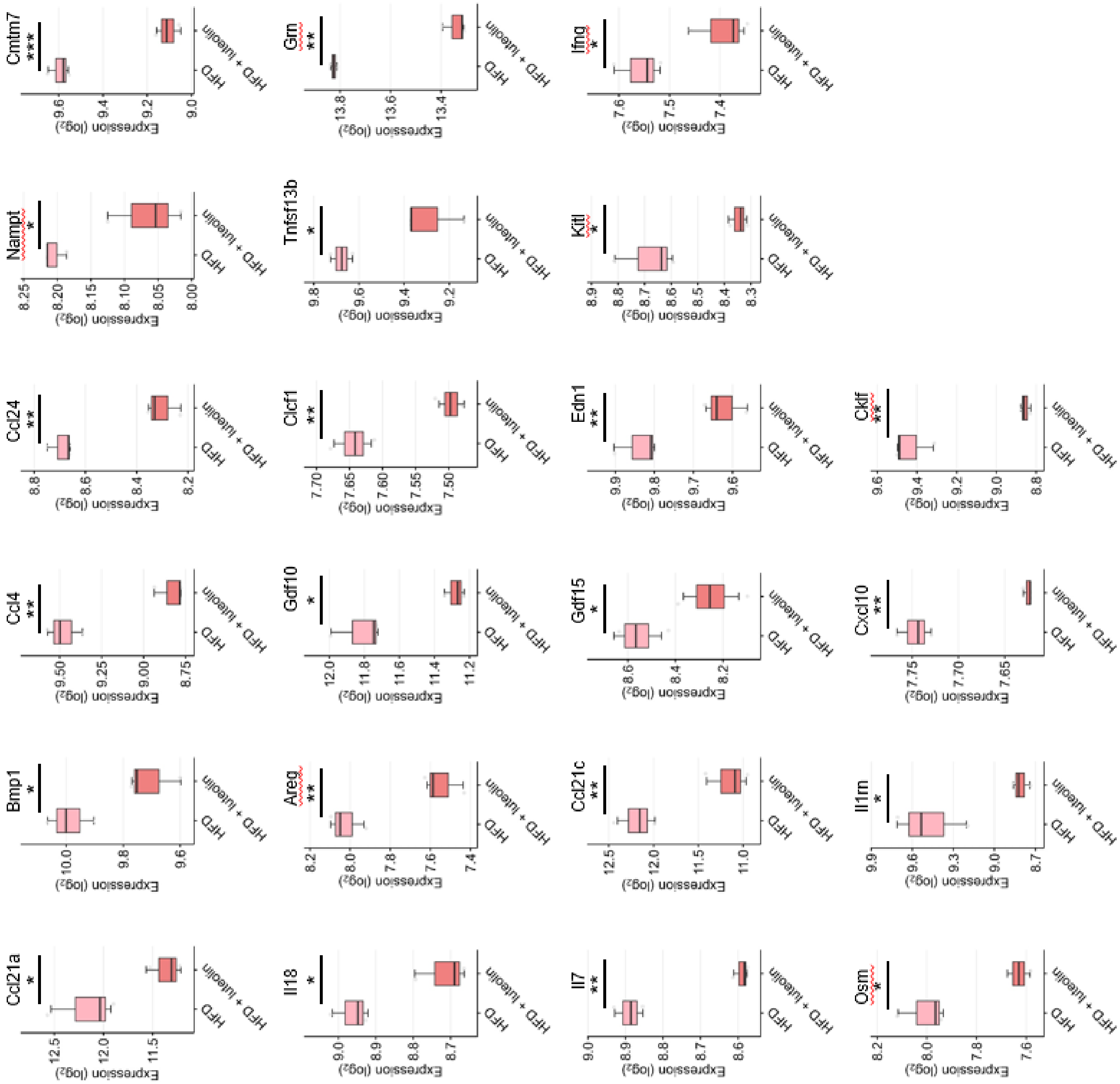

Supplementary Figure S4. GSE111412. Comparison of Ccl21a, Bmp1, Ccl4, Ccl24, Nampt, Cmtm7, Il18, Areg, Gdf10, Clcf1, Tnfsf13b, Grn, Il7, Ccl21c, Gdf15, Edn1, Kitl, Ifng, Osm, Il1rn, Cxcl10, and Cklf expression in HFD- and HFD + luteolin-treated mouse epididymal adipose tissue. The gene expression of each cytokine/chemokine was analyzed using the GSE111412 dataset. ***: $p < 0,001$, **: $p < 0.01$, and *: $p < 0.05$ compared to the induction group.
